# A Case of Ulceration in the Stomach

**Published:** 1800-06

**Authors:** James Moore

**Affiliations:** Grosvenor Street


### *A Case of Ulceration in the Stomach.*

A Stout middle-aged woman had long complained of conſiderable pain in her ſtomach, which ſometimes darted through to her back; ſhe likewiſe had occaſional fits of vomiting in the morning. Theſe ſymptoms were attributed by her friends to drinking, to which ſhe was much addicted; the event, however, makes it more probable, that her ſufferings were the cauſe of her drinking.

She paſſed the morning of the 29th of March laſt at an alehouſe; and, at one o'clock in the forenoon, ſhe was attacked ſuddenly with ſuch a violent pain all over her belly, as to be forced to ſcream aloud.

Mr. Patten was immediately called to her aſſiſtance, who employed ſuch remedies as he judged proper. He informed me, that when he ſaw her, her pulſe was quick and weak; and ſome degree of coldneſs had taken place on the extremities.

The ſufferings of the patient abated with her ſtrength; ſhe ſunk rapidly, and expired thirteen hours after ſhe was attacked with the pain.

I opened the body thirty hours after her death, and although the corpſe lay in a room without a fire, yet the abdomen was diſtended with gas, and there was ſome emphyſema in the cellular membrane, the effect of beginning putrefaction.—This evinces that gin and porter, though drank plentifully during life, have little power in retarding putrefaction after death.

The firſt preternatural circumſtance which occurred in opening the abdomen, was the effuſion of about two quarts of a whitiſh fluid. It was diſcovered, that the ſource of this was a circular orifice in the ſtomach, about a quarter of an inch in diameter; and it appeared that the white fluid was gruel and the other drinks, which the patient had ſwallowed previous to her diſeaſe, mingled with the ſecretions of the ſtomach. Upon examining the internal ſurface of this organ, two ulcers were diſcovered, each about an inch in length, of an oval form, and apparently ſpreading towards each other. In the centre of one of the ulcers was the ſmall hole formerly mentioned, its edge was thin and ſmooth; the ſubſtance of the ſtomach, near the ulcers, was thickened, and in ſome degree inflamed; the peritoneum was ſlightly inflamed, and the body had no other diſeaſed appearance.

The diſeaſe which deſtroyed this poor woman, though uncommon, has been mentioned by authors. Bonetus, Morgagni, as well as others, have recorded ſimilar caſes; and the various

 appearances appearances of ulcers in the ſtomach, are accurately deſcribed in Dr. Baillie's Morbid Anatomy.

This is, probably, a more frequent cauſe of ſudden death than is generally imagined, for it is the ſecond inſtance I have met with.—The firſt was a very young girl, whoſe only complaint was occaſionally vomiting her food. This gave her ſo little uneaſineſs, that ſhe tried as much as poſſible to conceal it, leſt ſhe ſhould be adviſed to ſwallow medicines. One night ſhe was ſeized with what was believed to be a very violent fit of the colic. Opiates did not diminiſh the pain; in a few hours cold ſweats broke out, her pain left her, and ſhe died in ſixteen hours from the beginning of the attack.

Two circumſtances occurred in both theſe caſes, different, I think, from what was naturally to be expected.

The firſt is, the ſudden attack of excruciating pain, which was felt all over the belly. I know no alteration in the diſeaſed parts which could have occurred to produce this effect, except the opening in the peritoneal coat of the ſtomach, and the effuſion of its contents into the general cavity of the abdomen.

The gaſtric juice gives no ſenſation to the ſtomach itſelf; but it is, perhaps, capable of exciting all the torture theſe patients endured, when applied to the peritoneum, a membrane not adapted by Nature to ſuſtain its application.

The ſecond circumſtance is the very ſudden death of the patients;—to what is this to be attributed? The moſt ignorant medical man might eaſily have foretold, that theſe patients could not recover after a hole was formed in their ſtomachs; but, I doubt if the wiſeſt could have propheſied that this event would put ſo ſpeedy a termination to life. The ſymptoms of this malady are few and equivocal. But if it could be known, that any one had an ulcer in the ſtomach previous to its penetrating into the abdomen, a regimen and treatment might be preſcribed, which, poſſibly, would contribute to heal it. For it is certain, that ulcers in this organ have healed; poiſons have been ſwallowed, which muſt have eroded portions of the internal ſurface of the ſtomach, and wounds have been received into it without proving mortal.

The chance of curing this diſeaſe being, however, very ſmall, let us turn our attention towards the cauſes and the prevention of ſo dangerous a diſtemper: we muſt here, as in other parts of the obſcure ſcience of medicine, have recourſe to analogy and conjectures. Ulcers upon the external parts of the body, are produced either by diſeaſes or by accidents. The latter is the more common cauſe; and this may likewiſe be the caſe with ulcers in the ſtomach.

Many perſons are extremely raſh in ſwallowing fiſh bones, fruit fruit ftones, and other hard and fharp fubftances. Women frequently fwallow pins without fear, fo that it feems to me very difficult to give a good reafon, why ulcers in the ftomach occur fo feldom as they do. It is to be wifhed, that the danger of fuch practices was more generally inculcated, that a real benefit might refult from thefe diffections; for it is known to all furgeons, that a very flight puncture in the fkin fometimes degenerates into an ill-conditioned ulcer.

The ftomach is not invulnerable, and it is fufceptible of ulceration, as well as the fkin. The contact of the gaftric juice, and the variety of foods which are fwallowed together, with the action of the ftomach, are not very favourable circumftances for healing an injury in this part; and fhould any of thefe circumftances, or fome malady in the conftitution, excite ulceration, a healing difpofition may never take place; and, if the ulcer fpreads and pierces the coats of the ftomach, a fudden and painful death is the inevitable confequence.

*Grofvenor Street,*

*April* 23, 1800.

JAMES MOORE.

